# Supplementary material for: Serum Mac-2 Binding Protein Levels Associate with Metabolic Parameters and Predict Liver Fibrosis Progression in Subjects with Fatty Liver Disease: A 7-Year Longitudinal Study
Source: Nutrients. 2020 Jun 12;12(6):1770. doi: 10.3390/nu12061770 (PMC7353396; doi:10.3390/nu12061770)
Supplement: Supplementary file 1 [file nutrients-12-01770-s001.pdf]

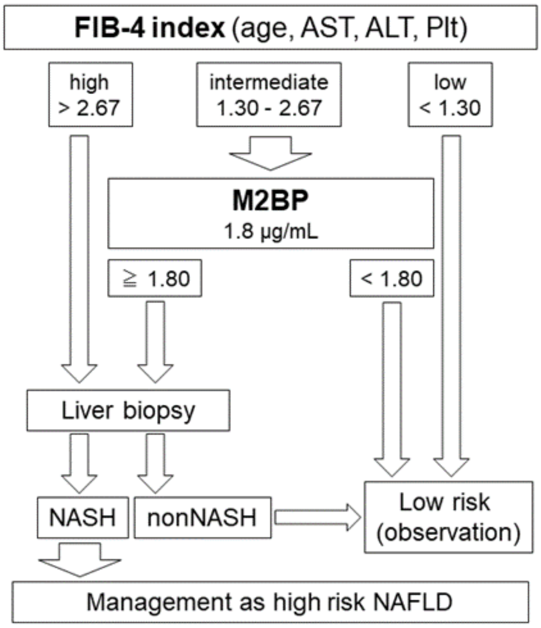

Figure S1. Proposed management strategy algorithm for nonalcoholic fatty liver disease. Based on the results of our study, we propose a management protocol for NAFLD using the FIB4-index and serum M2BP levels.

Table S1. Relationship between serum M2BP levels and metabolic risk factors in male and female.

(A) Comparisons between serum M2BP levels and each metabolic syndrome–related disease at baseline in male

**Disease**	**Positive**	**Negative**	***P* value**
Obesity	1.78 ± 0.97	1.26 ± 0.88	<0.0001
Hypertension	1.75 ± 1.07	1.35 ± 0.84	<0.0001
Dyslipidemia	1.73 ± 1.06	1.33 ± 0.82	<0.0001
Diabetes mellitus	1.59 ± 1.00	1.38 ± 0.87	<0.01
Fatty liver	1.73 ± 1.00	1.01 ± 0.60	<0.0001

(B) Comparisons between serum M2BP levels and each metabolic syndrome–related disease at baseline in female

**Disease**	**Positive**	**Negative**	***P* value**
Obesity	2.30 ± 2.41	1.34 ± 1.01	<0.0001
Hypertension	2.05 ± 1.25	1.52 ± 1.70	<0.0001
Dyslipidemia	1.95 ± 1.35	1.54 ± 1.70	<0.005
Diabetes mellitus	1.75 ± 2.10	1.53 ± 1.08	N.S.
Fatty liver	2.42 ± 2.32	1.13 ± 0.62	<0.0001

(C) Multiple logistic regression analysis of factors associated with serum M2BP levels at baseline in male

**Factor**	**t value**	***P* value**	**95% CI**	
			**Lower**	**Upper**
Obesity (*y*=1, *n*=2)	3.22	<0.005	0.0532	0.219
Hypertension (*y*=1, *n*=2)	2.89	<0.005	0.0375	0.197
Dyslipidemia (*y*=1, *n*=2)	1.86	N.S.	-0.00444	0.157
Diabetes mellitus (*y*=1, *n*=2)	2.06	<0.05	0.00388	0.165
Fatty liver (*y*=1, *n*=2)	5.57	<.0001	0.169	0.353

(D) Multiple logistic regression analysis of factors associated with serum M2BP levels at baseline in female

**Factor**	**t value**	***P* value**	**95% CI**	
			**Lower**	**Upper**

Obesity (*y*=1, *n*=2)	0.76	N.S.	-0.186	0.422
Hypertension (*y*=1, *n*=2)	-0.32	N.S.	-0.369	0.266
Dyslipidemia (*y*=1, *n*=2)	-0.63	N.S.	-0.398	0.205
Diabetes mellitus (*y*=1, *n*=2)	0.12	N.S.	-0.218	0.246
Fatty liver (*y*=1, *n*=2)	3.93	<0.0001	0.311	0.94

Table S2. Multivariate analysis of predicted changes in NFS using baseline various variables

**Variable**	**t value**	***P* value**	**95% CI**	
			**Lower**	**Upper**
Gender (F)	-1.14	N.S.	-0.145166	0.038823
Age	2.97	<0.005	0.004246	0.020766
BMI (kg/m^2)	1.57	N.S.	-0.003543	0.032138
Alcohol consumption (g/week)	0.47	N.S.	-0.000408	0.000664
SBP (mm Hg)	0.49	N.S.	-0.003056	0.005063
ALT (U/L)	4.05	<0.05	0.268913	0.77524
GGT (U/L)	-0.38	N.S.	-0.247491	0.166625
T-Bil (mg/dL)	2.1	N.S.	0.000202	0.006184
Albumin (mg/dL)	0.64	<0.0001	-0.000568	0.001122
Creatinine (mg/dL)	0.17	N.S.	-0.000893	0.001059
CHE (U/L)	-1.62	N.S.	-0.876249	0.084667
TG (mg/dL)	1.09	<0.005	-0.00079	0.002774
T-Chol (mg/dL)	-1.41	N.S.	-0.00112	0.000182
Uric acid (mg/dL)	-0.86	N.S.	-0.075527	0.029571
Iron (μg/dL)	-7.36	<0.0001	-0.007909	-0.004577
FBG (mg/dL)	-0.8	N.S.	-0.00493	0.002087
HbA1c (%)	2.12	<0.05	0.008548	0.221612
M2BP (μg/mL)	2.7	<0.01	0.019779	0.124948

Table S3. Multivariate analysis of predicted changes in serum M2BP levels using changes in various variables by gender

(A) Multivariate analysis of predicted changes in serum M2BP levels using changes in various variables in male.

Variable	t value	*P* value	95% CI	
			Lower	Upper
ΔBMI (kg/m²)	0.88	N.S.	-0.0452	0.118
Δalcohol consumption (g/week)	0.5	N.S.	-0.000866	0.00146
ΔSBP (mm Hg)	1.98	<0.05	3.51E-05	0.0123
ΔALT (U/L)	2.04	<0.05	0.000192	0.0099
ΔGGT (U/L)	2.54	<0.05	0.000489	0.00382
ΔAlbumin (mg/dL)	0.37	N.S.	-0.405	0.593
ΔCHE (U/L)	1.33	N.S.	-0.000998	0.00517
ΔTG (mg/dL)	-0.37	N.S.	-0.00132	0.000899
ΔT-Chol (mg/dL)	0.97	N.S.	-0.00163	0.00481
ΔUric acid (mg/dL)	-0.98	N.S.	-0.139	0.0466
ΔCreatinine (mg/dL)	0.58	N.S.	-0.314	0.577
ΔFBG (mg/dL)	0.33	N.S.	-0.00408	0.00575
ΔHbA1c (%)	0.96	N.S.	-0.0903	0.264
ΔIron (μg/dL)	-1.3	N.S.	-0.00357	0.000721
ΔPlatelet count (× 10^4/μL)	0.34	N.S.	-0.0259	0.0368

(B) Multivariate analysis of predicted changes in serum M2BP levels using changes in various variables in female.

Variable	t value	*P* value	95% CI	
			Lower	Upper
ΔBMI (kg/m²)	-0.01	N.S.	-0.11	0.109
Δalcohol consumption (g/week)	-0.53	N.S.	-0.00634	0.00366

ΔSBP (mm Hg)	-0.21	N.S.	-0.014	0.0113
ΔALT (U/L)	-3.03	<0.005	-0.0267	-0.00559
ΔGGT (U/L)	0.92	N.S.	-0.00299	0.00817
ΔAlbumin (mg/dL)	0.25	N.S.	-0.978	1.25
ΔCHE (U/L)	2.08	<0.05	0.000224	0.00912
ΔTG (mg/dL)	1.3	N.S.	-0.00185	0.00888
ΔT-Chol (mg/dL)	0.23	N.S.	-0.00599	0.00754
ΔUric acid (mg/dL)	-0.3	N.S.	-0.317	0.234
ΔCreatinine (mg/dL)	-0.11	N.S.	-3.13	2.79
ΔFBG (mg/dL)	-1.1	N.S.	-0.0206	0.00584
ΔHbA1c (%)	1.74	N.S.	-0.0437	0.686
ΔIron (μg/dL)	-0.93	N.S.	-0.00633	0.00228
ΔPlatelet count ($\times 10^4$/μL)	0.76	N.S.	-0.0293	0.0658
